# Procedural memory consolidation is associated with heart rate variability and sleep spindles

**DOI:** 10.1111/jsr.12910

**Published:** 2019-08-27

**Authors:** Frank J. van Schalkwijk, Theresa Hauser, Kerstin Hoedlmoser, Mohamed S. Ameen, Frank H. Wilhelm, Cornelia Sauter, Gerhard Klösch, Doris Moser, Georg Gruber, Peter Anderer, Bernd Saletu, Silvia Parapatics, Josef Zeitlhofer, Manuel Schabus

**Affiliations:** ^1^ Laboratory for Sleep Cognition and Consciousness Research Centre for Cognitive Neuroscience (CCNS) University of Salzburg Salzburg Austria; ^2^ Clinical Stress and Emotion Laboratory Division of Clinical Psychology, Psychotherapy and Health Psychology Department of Psychology University of Salzburg Salzburg Austria; ^3^ Department of Neurology Medical University of Vienna Vienna Austria; ^4^ Competence Center of Sleep Medicine Charité – University Medicine Berlin Germany; ^5^ Department of Psychiatry and Psychotherapy Medical University of Vienna Vienna Austria

**Keywords:** HRV, mirror tracing, memory, motor skill adaptation, polysomnography, spindles

## Abstract

Sleep and memory studies often focus on overnight rather than long‐term memory changes, traditionally associating overnight memory change (OMC) with sleep architecture and sleep patterns such as spindles. In addition, (para‐)sympathetic innervation has been associated with OMC after a daytime nap using heart rate variability (HRV). In this study we investigated overnight and long‐term performance changes for procedural memory and evaluated associations with sleep architecture, spindle activity (SpA) and HRV measures (R‐R interval [RRI], standard deviation of R‐R intervals [SDNN], as well as spectral power for low [LF] and high frequencies [HF]). All participants (*N *=* *20, *M*_age_ = 23.40 ± 2.78 years) were trained on a mirror‐tracing task and completed a control (normal vision) and learning (mirrored vision) condition. Performance was evaluated after training (R1), after a full‐night sleep (R2) and 7 days thereafter (R3). Overnight changes (R2‐R1) indicated significantly higher accuracy after sleep, whereas a significant long‐term (R3‐R2) improvement was only observed for tracing speed. Sleep architecture measures were not associated with OMC after correcting for multiple comparisons. However, individual SpA change from the control to the learning night indicated that only “SpA enhancers” exhibited overnight improvements for accuracy and long‐term improvements for speed. HRV analyses revealed that lower SDNN and LF power was associated with better OMC for the procedural speed measure. Altogether, this study indicates that overnight improvement for procedural memory is specific for spindle enhancers, and is associated with HRV during sleep following procedural learning.

## 1 INTRODUCTION

Following overnight memory changes (OMC), additional long‐term changes can occur over subsequent nights of sleep (Walker et al., 2003). Yet relatively few studies have investigated long‐term effects of healthy, undisturbed sleep on memory performance. Instead, memory performance is contrasted between healthy sleep and (partial) sleep deprivation directly after learning and after 48–72 hr. Upon recovery, the sleep group generally shows better memory recall compared to the sleep‐deprivation group for declarative word‐pair learning (Gais et al., 2007) and foreign language vocabulary learning (Gais, Lucas, & Born, 2006). Yet, Schönauer, Gratsch, and Gais (2015) found better memory recall for declarative word‐pairs after sleep compared to sleep deprivation after the first night, whereas no differences were observed after three recovery nights. For procedural memory, the sleep group generally shows better long‐term performance compared to the sleep‐deprivation group on a motor adaptation task after 3 days (Maquet, Schwartz, Passingham, & Frith, 2003; Schönauer et al., 2015) or on a visual discrimination task after 7 days (Stickgold, James, & Hobson, 2000). Crucially, these studies compared memory performance after sleep with that after sleep deprivation and were not specifically aimed at evaluating the relevance of sleep for long‐term memory consolidation.

Traditionally, sleep studies investigate associations between OMC and full‐night sleep (cf., Diekelmann & Born, 2010; Fogel, Smith, & Cote, 2007; Gais et al., 2006; Walker et al., 2003), but have increasingly evaluated associations between OMC and sleep characteristics such as sleep spindle properties (e.g., spindle density [spindles/min; SpD] and activity [amplitude x duration; SpA]). Increased SpA during the learning night compared with the control night was found to be positively associated with OMC for word‐pair recall (Schabus et al., 2004). In addition to these investigations, heart rate variability (HRV) is a new area of interest when evaluating memory and cognitive performance. HRV can be assessed for the time domain by using the R‐R interval (RRI) and standard deviation of R‐R intervals (SDNN), as well as for the frequency domain by using spectral power for low (LF; 0.04–0.15 Hz) and high frequencies (HF; 0.15–0.40 Hz). Although the association between LF and sympathetic activity is debated (cf., Del Paso, Langewitz, Mulder, Van Roon, & Duschek, 2013; Trinder et al., 2001), the HF component has been well validated as a measure of parasympathetic activity (Berntson et al., 1997) and has been related to cognitive performance (Thayer, Hansen, Saus‐Rose, & Johnsen, 2009), executive functioning and working memory (Cellini, De Zambotti, Covassin, Sarlo, & Stegagno, 2014; Hansen, Johnsen, Sollers, Stenvik, & Thayer, 2004; Hansen, Johnsen, & Thayer, 2003). Considering the positive associations between HF and cognitive performance during wakefulness (Hansen et al., 2003; Luque‐Casado, Zabala, Morales, Mateo‐March, & Sanabria, 2013), HRV and specifically HF may be an additional variable of interest when investigating sleep‐dependent memory consolidation. Although sleep studies generally implement heart rate measurements, few have analyzed its association with memory. Using daytime nap protocols, Whitehurst, Cellini, Mcdevitt, Duggan, and Mednick (2016) found HF during rapid eye movement (REM) to be positively associated with performance improvement on a declarative remote associates test, whereas Naji, Krishnan, Mcdevitt, Bazhenov, and Mednick (2019) found HF during N2 to be positively correlated with improvement on a declarative face–name association task. Investigations for full‐night sleep protocols as well as the procedural memory domain are as of yet lacking, yet longer sleep duration may provide a clearer insight into HRV during sleep and its relation to OMC.

The present study evaluated potential long‐term (7 days) memory performance changes subsequent to OMC and investigated associations of SpA and HRV with OMC with regard to procedural memory. This study was part of a larger project that evaluated the effects of full‐night sleep on the consolidation of declarative and procedural information. Associations between sleep, SpA and OMC have been published for the declarative memory domain (Schabus et al., 2004). OMC for the procedural memory domain have been compared to findings from a daytime nap study that replicated the full‐night study protocol. Results indicate that daytime naps prevent the memory deterioration observed during an identical period of daytime wakefulness, whereas full‐night sleep may result in significant improvements in performance (van Schalkwijk et al., 2019). Following the recent studies on HRV and OMC during a daytime nap (Naji et al., 2019; Whitehurst et al., 2016), the present study re‐analyzed the procedural dataset of the full‐night study to investigate long‐term (7 days) memory performance changes and associations between SpA and HRV as potential mediators of OMC that are underrepresented in the current literature. Participants were trained on a procedural mirror‐tracing task for which short‐ (12 hr) and long‐term (7 days) performance changes were evaluated. Overnight and long‐term (7 days) improvements were expected. Enhancement in SpA from the control to the learning conditions was expected to be positively associated with performance improvements, as found earlier for the declarative domain (Schabus et al., 2004). HF was expected to be positively associated with OMC, as recently found for the declarative domain (Naji et al., 2019; Whitehurst et al., 2016).

## 2 METHODS

### 2.1 Participants

The initial sample (*N *=* *24) were participants trained on a procedural memory task as part of a larger study investigating OMC for procedural and declarative memory domains (Schabus et al., 2004; *N *=* *48, 50% male). Participants had no sleep disorders, anxiety disorders or major depression, and did not take medication or drugs that could have influenced the study outcomes (for details, see Schabus et al., 2004). Four participants were excluded due to behavioural performance (*M *±* *3 *SD*;* n *=* *1), low sleep efficiency (<80%; *n *=* *1) or long sleep onset latency (>20 min; *n *=* *2). The final sample consisted of 20 right‐handed participants (*M*_age_ = 23.40 ± 2.78 years, range = 20–30 years; 45% male) who provided informed consent prior to protocol onset. The study was approved by the university ethics committee.

### 2.2 Instruments

#### 2.2.1 Procedural memory task

Participants were trained on a procedural mirror‐tracing paradigm (Plihal & Born, 1997) with instructions to retrace 12 stimuli as quickly and accurately as possible over 12 separate 90‐s trials (see Figure S1). Outcome variables were the length of the trace (speed), number of errors and the percentage of time that the trace deviated from the stimulus line (error time [%]). The control task used an identical paradigm and instructions, yet used different stimuli and allowed direct vision of the stimuli. As regular tracing was regarded as straightforward, only the number of errors and error time were evaluated.

#### 2.2.2 EEG and polysomnography

Recordings were conducted using 21 scalp electrodes and the Neuroscan system (NeuroScan Inc., El Paso, Texas). Placement of the electrodes was carried out according to the international 10/20 system (Fp1, Fpz, Fp2, F3, Fz, F4, F7, F8, T3, C3, Cz, C4, T4, T5, P3, Pz, P4, T6, O1, Oz, O2, and additional mastoids A1 and A2). The polysomnography (PSG) set‐up included one bipolar electrocardiogram electrode, one bipolar respiratory channel, one bipolar submental electromyogram electrode and five electrooculogram electrodes. Data were acquired using a 250‐Hz sampling rate and online filters (high pass = 0.10 Hz; low pass = 70 Hz; 50 Hz Notch filter).

### 2.3 Procedures

**Figure 1:**
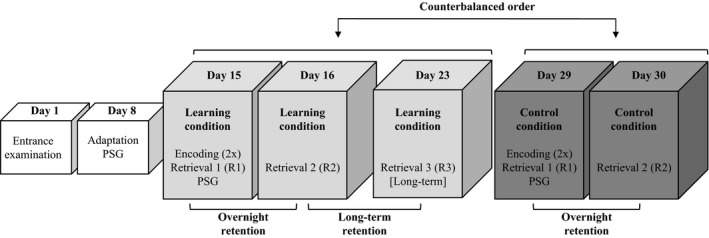
Study protocol. Participants were screened prior to inclusion. An adaptation polysomnography (PSG) was conducted 1 week preceding the first PSG. Learning and control conditions were counterbalanced between participants. Overnight memory changes (R2‐R1) were evaluated for both conditions, whereas long‐term performance changes (R3‐R2) were only assessed for the learning condition

The 30‐day study protocol (Figure 1) started with an entrance examination for exclusion criteria (Day 1) and an adaptation PSG (Day 8) to account for first‐night effects. Participants were randomly assigned to a counterbalanced condition order. The evening encoding session (Day 15) included training on the mirror‐tracing task (or respective control task) during two succeeding blocks, after which performance was evaluated (retrieval 1; R1). Retrieval and subsequent PSG onset were separated by 1 hr, with bedtimes and PSG onset between 23:00 and 00:00 hours. PSG recordings were stopped after reaching the individual habitual sleep duration or 8 hr of sleep. The second performance evaluation (retrieval 2; R2) was conducted 1 hr after awakening to account for sleep inertia. This procedure was repeated for the second part of the protocol for either control or learning conditions, depending on condition order. Only the learning condition included a long‐term (7 days) follow‐up (retrieval 3; R3). Each session enquired about mood, drive, affectivity and fatigue using 100‐mm visual analogue scales (ASES; Ott, Oswald, Fichte, & Sastre‐Hernandez, 1986; see Figure S2).

### 2.4 Analyses

Performance changes were evaluated for *overnight* (R2‐R1) and *long‐term* (R3‐R2) retention. Importantly, the speed variable was not evaluated for six participants during the learning condition due to technical problems. Consequently, the analyses that utilized the speed variable used a lower sample of participants (*n *=* *14). Sleep scoring was automatically conducted using the Somnolyzer (The Siesta Group, Vienna, Austria) following standard criteria (Rechtschaffen & Kales, 1968). Automated sleep scoring was visually reviewed and corrected if necessary. Subsequent automated detection of sleep spindles was conducted using custom scripts for channels C3 and C4 following previously described criteria for spindle amplitude (≥12 μV), duration (0.3–2.0 s) and frequency range (11–16 Hz; Anderer et al., 2005). Detected spindles were further evaluated by a linear discriminant analysis that had been trained on visually detected spindles. A last classification was carried out based on frequency ranges for slow (11–13 Hz) and fast spindles (13–15 Hz).

The electrocardiogram (ECG) data were low‐pass filtered (30 Hz), which was followed by automatic R‐peak detection using BrainVision Analyzer 2.1.1.327 (Brain Products GmbH) with the ‘ECG Markers’ solution. R‐peak markers were visually verified and corrected where necessary. Careful manual artifact correction was conducted over the whole night to account for major artifacts such as body movements. Each PSG recording was segmented by stage (Wake, N1, N2, N3 and REM), as indicated by the automated sleep scoring. Note that the wake classification includes both wakefulness prior to sleep as well as wake after sleep onset. RR interval time series were extracted per segmented wake and sleep stage (continuous 2‐min segments). Note that the use of continuous 2‐min segments led to a loss of data on sleep stages that were of shorter duration (14% of the total dataset). Furthermore, segments containing body movement or artefactual RR intervals were removed (12% of the segmented data). Thus, 76% of the total dataset was used for HRV analyses. HRV data were computed per segment using the software Autonomic Nervous System Laboratory (ANSLAB) version 2.6 (Blechert, Peyk, Liedlgruber, & Wilhelm, 2016; time domain: mean RR interval [RRI] and SDNN; frequency domain: spectral powers for low‐frequency [LF; 0.04–0.15 Hz] and high‐frequency [HF; 0.15–0.40 Hz]). HRV measures were subsequently averaged per sleep stage and all spectral power values were normalized by natural logarithm.

Statistical analyses were carried out with IBM SPSS Statistics version 24 (Armonk, NY, USA). Sleep architecture was contrasted between conditions (Wilcoxon), whereas associations between OMC, sleep architecture and spindle characteristics were investigated using Spearman correlations. HRV measures were contrasted between sleep stages using repeated measures ANOVAs (four levels: N1, N2, N3 and REM) followed by Wilcoxon post‐hoc tests. Greenhouse‐Geisser corrections were applied when assumptions of sphericity were violated as indicated by Mauchly's test of sphericity. Note that wakefulness was not included in the HRV analyses as no wake segments were available for a large number of participants (*n *=* *12), nor was it of main interest for this project. Although wake classification has been included in the figures and tables as rough estimates, these representations should be considered with caution. Group contrasts were conducted using Mann–Whitney *U*‐tests. Results report mean ± *SD*. Effect size estimates and 95% confidence intervals (CI) were calculated in R version 3.5.1 using the “effsize” v0.7.4 (Torchiano, 2018) and “metaphor” v2.0‐0 (“escalc” function; Viechtbauer, 2017) packages.

## 3 RESULTS

### 3.1 Memory performance changes

#### 3.1.1 Control condition

Only *overnight memory changes* were evaluated for error number and error time, showing an overnight reduction for the number of errors from R1 (11.66 ± 26.89) to R2 (5.33 ± 10.51; *Z *=* *−2.25, *p *=* *.025, *d *=* *0.37, 95% CI [−0.13, 0.83]). As previously reported (van Schalkwijk et al., 2019), error time also showed an overnight reduction from R1 (1.27 ± 3.43) to R2 (0.41 ± 1.08; *Z *=* *−2.373, *p *=* *.018, *d *=* *0.68, 95% CI [0.05, 2.25]).

#### 3.1.2 Learning condition

As previously reported (van Schalkwijk et al., 2019), *overnight memory changes* showed no significant change for speed from R1 (119.99 ± 60.14) to R2 (113.67 ± 64.33; *Z *=* *−0.28, *p *=* *.778). Yet the number of errors reduced from R1 (7.87 ± 18.70) to R2 (5.79 ± 14.50; *Z *=* *−3.14, *p *=* *.002, *d *=* *0.47, 95% CI [−0.01, 0.91]), in addition to a reduction in error time from R1 (2.90 ± 6.09) to R2 (1.93 ± 4.71; *Z *=* *−2.06, *p *=* *.039, *d *=* *0.38, 95% CI [0.10, 2.75]).

*Long‐term performance changes* revealed an increase in speed from R2 to R3 (131.63 ± 83.35; *Z *=* *−2.86, *p *=* *.004, *d *=* *−0.89, 95% CI [−1.45, −0.23]). In contrast, no performance changes were observed for the number of errors from R2 to R3 (5.97 ± 15.61; *Z *=* *−0.11, *p *=* *.913) or for error time from R2 to R3 (1.59 ± 4.72; *Z *=* *−1.79, *p *=* *.073).

### 3.2 Sleep characteristics and performance changes

Following corrections for multiple comparisons (α = 0.016), no correlations were found between OMC and sleep architecture, nor for sleep architecture changes from control to learning conditions (all *p *≥* *.022). In addition, a long‐term (R3‐R2) increase in the number of errors was positively correlated with a higher percentage of N3 sleep during the learning night (*r*_*s*_[19] = 0.545, *p *=* *.013). No other correlations were found between sleep architecture and long‐term performance changes (all *p *≥* *.050) (for sleep architecture, see Table S1).

### 3.3 Sleep spindle activity and overnight memory changes

**Figure 2:**
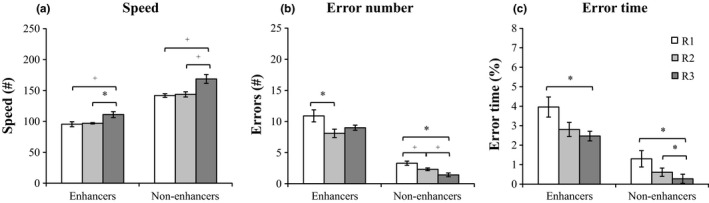
Behavioural performance illustrated for spindle enhancers and non‐enhancers (*M* ± *SEM*). Note that error bars have been adjusted to illustrate within‐subject variability. Performance changes were investigated per subgroup (spindle enhancers, *n *=* *12; spindle non‐enhancers, *n *=* *8) for (a) speed, (b) error number and (c) error time. Results indicate that only spindle activity (SpA) enhancers showed an overnight (R2‐R1) decrease in error number and long‐term (R3‐R2) increase in speed. No group differences were observed during any of the retrieval sessions on any performance measure. +*p *<* *.10, *p <* *.05

Participants were contrasted based on their change in SpA on channel C3 from control to learning conditions (spindle enhancers [change > 0; *n *=* *12]; spindle non‐enhancers [change ≤ 0; *n *=* *8]). Only spindle enhancers showed long‐term improvement in speed (*Z *=* *−2.31, *p *=* *.021, *d *=* *−0.82, 95% CI [−1.48, −0.002]; Figure 2a) and overnight reduction in error number (*Z *=* *−2.51, *p *=* *.012, *d *=* *0.50, 95% CI [−0.13, 1.07]; Figure 2b). No performance differences were observed between these subgroups on any performance measure during any of the retrieval sessions (all *p *≥* *.125). Note that the change in SpA was not associated with OMC for any performance measure (all *p *≥* *.328).

### 3.4 HRV during sleep

No differences in HRV measures were observed between control and learning nights across all sleep stages (see Table S2; all *p *≥* *.355). However, HRV measures differed between sleep stages during the learning condition (see Figure S3). A repeated measures ANOVA with the within‐subject factor sleep stage (N1, N2, N3, & REM) revealed significant within‐subject effects across stages for mean RRI (*F*_1.80, 34.23_ = 10.26, *p *<* *.001, $\eta_{p}^{2}$ = 0.35), SDNN (*F*_1.68, 31.98_ = 6.29, *p *=* *.007, $\eta_{p}^{2}$ = 0.25) and LF (*F*_1.78, 33.91_ = 9.26, *p *=* *.001, $\eta_{p}^{2}$ = 0.33), whereas no effect was observed for HF (*F*_3, 57_ = 1.16, *p *=* *.193, $\eta_{p}^{2}$ = 0.08). Post‐hoc contrasts between sleep stages were conducted using Wilcoxon signed ranks tests, which showed similar results to prior studies (Cellini, Whitehurst, Mcdevitt, & Mednick, 2016; De Zambotti et al., 2014; Whitehurst et al., 2016). Mean RRI was significantly higher during N2 compared to all other sleep stages (all *p *≤* *.033), whereas SDNN was significantly lower during N3 compared to all other sleep stages (all *p *≤* *.023). In addition, significantly lower LF power was observed during N3 compared to stages N2 (*Z *=* *−3.659, *p *<* *.001) and REM (*Z *=* *−3.509, *p *<* *.001), whereas no difference was observed compared to stage N1 (*Z *=* *−0.971, *p *=* *.332).

### 3.5 HRV contrasted for overnight improvers vs. non‐improvers

Following previous studies (Naji et al., 2019; Whitehurst et al., 2016), associations between OMC and HRV were investigated (See Table S3). Overnight memory change for speed was negatively associated with SDNN (*r*_*s*_[12] = −0.701, *p *=* *.005), LF (*r*_*s*_[12] = −0.723, *p *=* *.003) and HF (*r*_*s*_[12] = −0.600, *p *=* *.023) during REM, meaning that higher inter‐beat variability and LF power resulted in worsened OMC. LF power during REM was also found to be negatively associated with overnight memory change for the number of errors (*r*_*s*_[18] = −0.492, *p *=* *.028). Note that no further associations were observed between OMC and HRV measures for NREM sleep stages (all *p *≥* *.052), nor were any associations found between long‐term memory changes and HRV measures for any sleep stage (all *p *≥* *.051). Importantly, only the negative association between OMC for speed and LF power during REM remains significant after Bonferroni correction for multiple comparisons (α* *= 0.0042).

**Figure 3:**
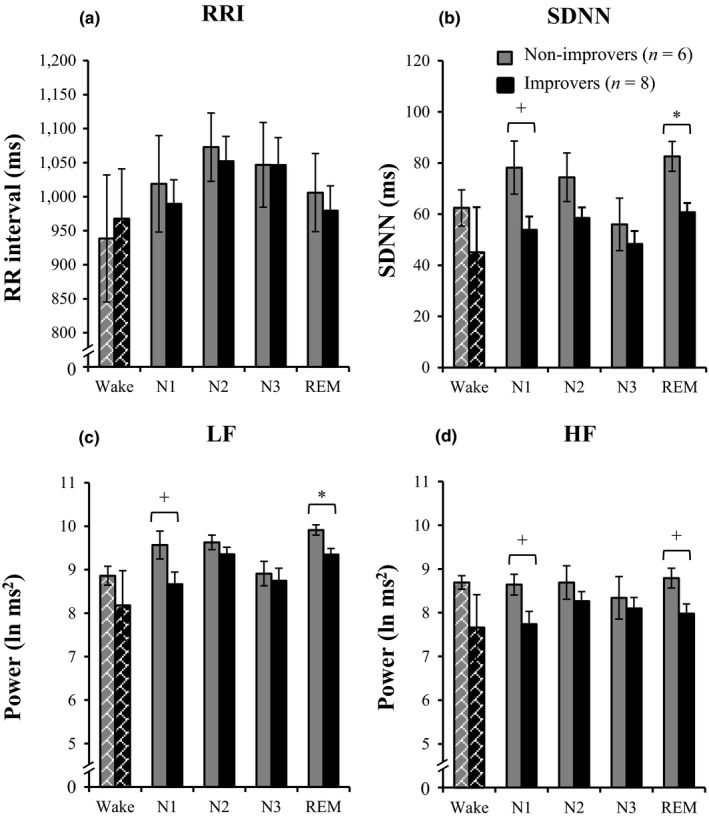
Post‐hoc contrasts on heart rate variability (HRV) characteristics between improvers and non‐improvers based on overnight memory change. Participants were contrasted based on the subdivision for overnight memory change (OMC) on the mirror‐tracing speed measure. No group differences were observed for (a) R‐R interval (RRI). (b) Standard deviation of R‐R intervals (SDNN) was found to be lower for improvers compared with non‐improvers during N1 and rapid eye movement (REM). (c) Power values were significantly lower for improvers during REM for low frequencies (LF) and (d) showed a trend for high frequencies (HF). Note that sufficient HRV data for wake were only available for a limited sample of participants (*n *=* *8) and should therefore be interpreted with caution. +*p *<* *.10; *p <* *.05

Following these correlations, we contrasted HRV measures between participants based on their overnight improvement for speed (improvers [change > 0; *n *=* *8]; non‐improvers [change ≤ 0; *n *=* *6]; Figure 3). We conducted a 2 × 4 repeated measures ANOVA (GROUP: improvers and non‐improvers; STAGE: N1, N2, N3 and REM), which revealed a main effect of group for SDNN (*F*_1, 12_ = 7.80, *p *=* *.016) and LF (*F*_1, 12_ = 4.84, *p *=* *.048). No effects were found for RRI (*F*_1, 12_ = 0.09, *p *=* *.775), HF (*F*_1, 12_ = 3.05, *p *=* *.107) or any of the interactions (all *p *≥* *.278). Exploratory post‐hoc contrasts (Figure 3) revealed that the main effect for SDNN is mainly driven by lower values for improvers compared with non‐improvers during N1 (*U *=* *10.00, *p *=* *.081) and REM (*U *=* *5.00, *p *=* *.013; Figure 3b). LF values were lower for improvers compared with non‐improvers during N1 (*U *=* *10.00, *p *=* *.081) and REM (*U *=* *7.00, *p *=* *.029; Figure 3c).

## 4 DISCUSSION

This study confirms that long‐term performance improvements over several days can take place in addition to OMC for procedural memory, contributing to prior observations (Maquet et al., 2003; Schönauer et al., 2015; Stickgold et al., 2000; Walker et al., 2003). Our results are aligned with those of Schönauer et al. (2015), who reported overnight reductions for mirror‐tracing error number and error time and no change for speed. Our results suggest that overnight consolidation of mirror tracing prioritizes accuracy over speed, and that consolidation of motor skill adaptation continues during subsequent nights, as previously shown by Walker et al. (2003). Categorizing participants based on a change in SpA from control to learning conditions revealed that only the SpA enhancers showed an overnight improvement for accuracy. This result extends the previously reported relevance of SpA enhancement for sleep‐dependent consolidation of declarative memory (Schabus et al., 2004) to the procedural memory domain.

Heart rate variability measures showed trends towards negative associations with OMC, specifically with SDNN and LF, indicating that memory improvement (or less forgetting) positively correlates with a lower SDNN and LF during sleep. This is contrary to previously reported positive associations between HF and cognitive performance during wake (Thayer et al., 2009) as well as associations between HF during a daytime nap and subsequent performance on declarative memory (Naji et al., 2019; Whitehurst et al., 2016). Yet, prefrontal activity has been closely linked with HF (Thayer & Lane, 2009; Thayer et al., 2009) as well as hippocampal activity during sleep, which might play an important role during “offline” systems consolidation of declarative information (Siapas & Wilson, 1998). Acquisition and consolidation of procedural memory, on the other hand, are considered less hippocampus dependent, which may explain why this study did not observe an association with HF. Rather, our observed negative associations with OMC seem to be largely driven by the LF component, which is under both sympathetic and parasympathetic control and is closely tied to cardiovascular adjustments due to the baroreflex (Goldstein, Bentho, Park, & Sharabi, 2011; Rahman, Pechnik, Gross, Sewell, & Goldstein, 2011). Previously, LF was considered as a measure of cardiovascular sympathetic activation, which for our results might have indicated that higher sympathetic activity during sleep could be detrimental to sleep‐dependent memory consolidation of procedural information. However, based on recent methodological evidence of LF being somewhat of a “mixed bag”, we must emphasize that such an interpretation may be too simplistic. In addition, OMC improvers indicated significantly lower SDNN. It could be speculated that only during wakefulness are higher variability and heart dynamics beneficial for processes such as working memory (Hansen et al., 2003), attention (Luque‐Casado et al., 2013) or general processes required to dynamically respond to a constantly changing environment.

As limitations we first need to mention the limited sample sizes of our subgroups and the lack of a wake control group. Yet, a wake control group would either require a period of daytime wakefulness, potentially introducing circadian effects, or sleep deprivation, potentially introducing differences due to fatigue, duration of prior wakefulness and sleep pressure. Therefore, we decided on a control condition of the same duration and with the same motor demand. Second, our PSG recordings did not include respiratory or physical activity measurements, which would be ideal to account for artifacts within the LF frequency range due to bouts of muscular activity (i.e., about every 7–25 s). Furthermore, respiratory effects on LF cannot entirely be excluded if some participants had a respiratory rate below 0.15 Hz, thus shifting respiratory sinus arrhythmia‐related HF to the LF band (Grossman & Taylor, 2007). Third, wake classification in the current study stems from the sleep recordings themselves and includes wakefulness during the sleep onset latency (SOL) period as well as wakefulness after sleep onset (WASO). It is important to note that the SOL period is usually characterized by an increased vagal drive, resulting in longer RRI as well as lower variability of breathing frequency and LF power (Shinar, Akselrod, Dagan, & Baharav, 2006). In contrast, WASO is more likely to reflect a transition state between sleep stages rather than real attentive wakefulness. Furthermore, such periods of arousal from sleep are associated with increased heart rate, blood pressure and sympathetic muscle activity (Guilleminault & Stoohs, 1995). As the wake state in this study contained periods of wakefulness from both sleep onset and wake after sleep onset, these results should be taken with caution. Considering these limitations, future studies on sleep and HRV are therefore encouraged to measure physical activity as well as respiration, and to include HRV evaluation during quiet seated pre‐sleep wakefulness in order to investigate associations of baseline HRV with possible memory trait effects.

In summary, our results demonstrate significant overnight and long‐term improvements for procedural memory performance. Furthermore, the overnight improvement was only evident for participants showing an increase in SpA from a control to a learning night, indicating the relevance of spindles also for procedural memory consolidation. The finding of LF being negatively associated with overnight procedural memory consolidation needs further attention as it is the first of its kind. For now we can only speculate that high LF and SDNN might be beneficial during waking but not during sleep as the brain and body should slow down and relax for successful offline consolidation in the absence of a need for dynamic regulation of incoming environmental stimuli.

## CONFLICT OF INTEREST

Research was funded by the Austrian Science Fund FWF project P‐15370. F.J. van Schalkwijk was additionally supported by the Doctoral College “Imaging the Mind” (FWF; W1233‐G17). The authors report no conflict of interests.

## AUTHOR CONTRIBUTION

CS, KH, PA, JZ and MS designed the study. KH, GK, MS, CS, SP and DM collected the data. TH, MSA, GG and FJVS processed the data. TH, FJVS and MS conducted statistical analyses. FHW contributed to the HRV analyses. FJVS and MS drafted the manuscript. CS, KH, GK, PA, BS, JZ and MS supervised the project. All authors commented on and edited the manuscript drafts.
