# Supplementary material for: Procedural memory consolidation is associated with heart rate variability and sleep spindles
Source: J Sleep Res. 2019 Aug 27;29(3):e12910. doi: 10.1111/jsr.12910 (PMC7317359; doi:10.1111/jsr.12910)
Supplement: Supplementary file 1 [file JSR-29-e12910-s001.docx]

**Supplementary material**

**Procedural memory consolidation is associated with heart rate variability and sleep spindles**

Frank J. van Schalkwijk^1^, Theresa Hauser^1^, Kerstin Hoedlmoser^1^, Mohamed S. Ameen^1^, Frank H. Wilhelm^2^, Cornelia Sauter^3,4^, Gerhard Klösch^3^, Doris Moser^3^, Georg Gruber^5^, Peter Anderer^5^, Bernd Saletu^5^, Silvia Parapatics^5^, Josef Zeitlhofer^3^, and Manuel Schabus^1,*^.

^1^ Laboratory for Sleep, Cognition and Consciousness Research, Centre for Cognitive Neuroscience [CCNS], University of

Salzburg, Salzburg, Austria.

^2^ Clinical Stress and Emotion Lab, Division of Clinical Psychology, Psychotherapy, and Health Psychology, Department of

Psychology, University of Salzburg, Salzburg, Austria.

^3^ Department of Neurology, Medical University Vienna, Vienna, Austria.

^4^ Competence Center of Sleep Medicine, Charité – University Medicine, Berlin, Germany.

^5^ Dept. of Psychiatry and Psychotherapy, Medical University of Vienna, Vienna, Austria.

^*^ Corresponding author.


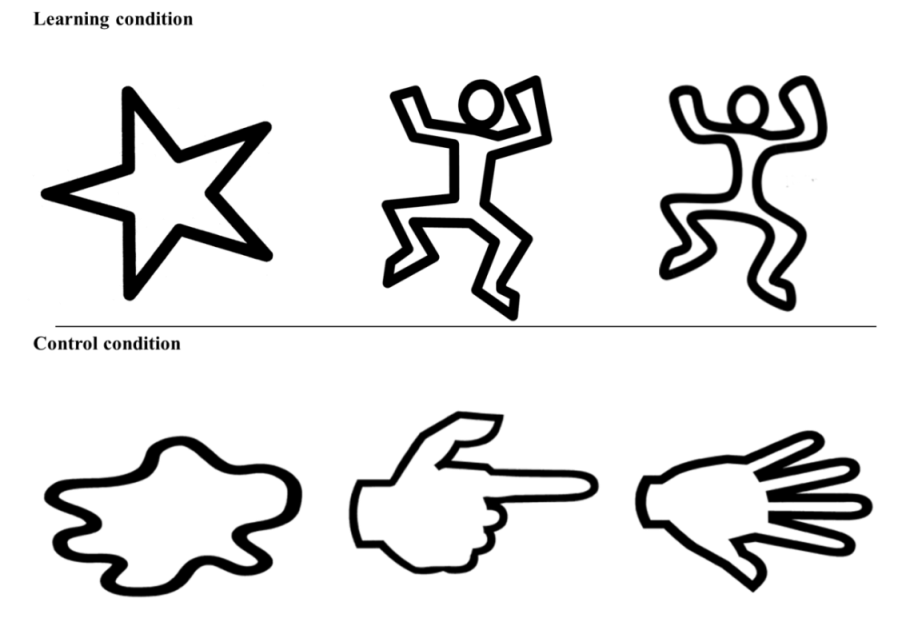


**Figure S1. Procedural mirror tracing task.** Participants were shown 12 stimuli and were instructed to retrace each stimulus as quickly and accurately as possible over 12 separate 90 s trials using an electronic stylus. Stimuli were only visible through a mirror as direct vision of the stimuli was prevented. Each encoding and retrieval session started with two dummy stimuli (left stimuli) and lasted ~30 min. The respective control task had participants trace 12 different stimuli for 90 s while stimuli were directly visible.

Subjective evaluations of drowsiness showed elevated drowsiness in the evening compared with the morning in both control, *t(*19) = 7.66, *p* < 0.001, *d* = 1.71, 95% CI [0.97, 2.32] and learning conditions, *t*(19) = 3.79, *p* = 0.001, *d* = 0.85, 95% CI [0.31, 1.32] (Supplementary Figure S2).


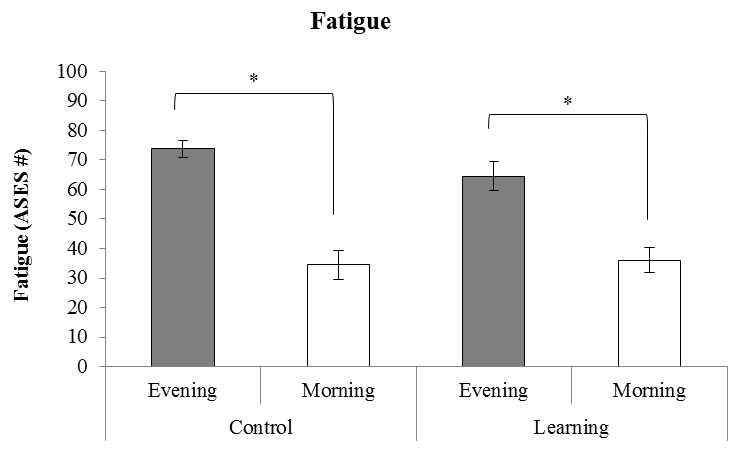


**Figure S2. Subjective evaluations of fatigue through ASES (M ± SEM).** A clear reduction in subjective fatigue was observed from evening to morning evaluations. Fatigue was reported using a 100-mm VAS-scale with 0 defined as “*Awake”* and 100 defined as “*Tired*”. No differences were observed in subjective fatigue between conditions for evening and morning reports. * *p* <0.05.

| **Table S1.** Sleep architecture during control and learning nights. | | | | |
| --- | --- | --- | --- | --- |
|  | CPSG | LPSG | *t*  value | *p*  value |
| Time in bed (min) | 487.45 ± 43.08 | 484.38 ± 36.15 | 0.43 | 0.675 |
| Total sleep time (min) | 467.80 ± 44.56 | 464.50 ± 38.59 | 0.50 | 0.626 |
| Efficiency (%) | 95.93 ± 2.33 | 95.85 ± 1.66 | 0.18 | 0.863 |
| N2 latency (min) | 14.63 ± 9.89 | 17.13 ± 11.41 | -0.92 | 0.369 |
| N1 (%) | 8.00 ± 4.17 | 8.11 ± 3.34 | -0.11 | 0.914 |
| N2 (%) | 55.79 ± 7.54 | 54.19 ± 8.63 | 1.36 | 0.190 |
| N3 (%) | 19.87 ± 5.42 | 20.94 ± 5.56 | -0.81 | 0.431 |
| REM (%) | 16.35 ± 4.97 | 16.38 ± 4.98 | -0.02 | 0.983 |
| Note: CPSG = control condition polysomnography; LPSG = learning condition polysomnography; REM = rapid eye movement sleep. | | | | |
|  | | | | |

| **Table S2.** Contrasting heart-rate variability (HRV) measurements per condition and sleep stage (*M* ± *SD*) | | | | | | |
| --- | --- | --- | --- | --- | --- | --- |
|  |  | **Wake** | **N1** | **N2** | **N3** | **REM** |
| **RRI**  **(ms)** | CPSG | 959 ± 120 | 997 ± 139 | 1041 ± 135 | 1017 ± 123 | 995 ± 144 |
|  | LPSG | 928 ± 155 | 1003 ± 131 | 1041 ± 113 | 1026 ± 125 | 982 ± 119 |
|  | *p*-values | 0.735 | 0.717 | 0.794 | 0.455 | 0.526 |
| **SDNN**  **(ms)** | CPSG | 66.14 ± 35.73 | 71.74 ± 25.70 | 66.49 ± 21.08 | 49.29 ± 19.59 | 73.86 ± 24.79 |
|  | LPSG | 52.73 ± 21.60 | 66.97 ± 27.65 | 66.22 ± 18.90 | 49.85 ± 19.53 | 72.26 ± 20.15 |
|  | *p*-values | 0.866 | 0.573 | 0.852 | 0.852 | 0.478 |
| **LF**  **(ln ms^2^)** | CPSG | 8.87 ± 1.26 | 9.26 ± 1.24 | 9.54 ± 0.57 | 8.66 ± 0.74 | 9.65 ± 0.74 |
|  | LPSG | 8.52 ± 0.87 | 9.11 ± 1.05 | 9.54 ± 0.56 | 8.73 ± 0.80 | 9.64 ± 0.67 |
|  | *p-*values | 0.612 | 0.841 | 0.940 | 0.911 | 0.881 |
| **HF**  **(ln ms^2^)** | CPSG | 8.11 ± 0.93 | 8.43 ± 1.01 | 8.40 ± 0.84 | 8.05 ± 0.81 | 8.27 ± 0.96 |
|  | LPSG | 8.06 ± 1.04 | 8.17 ± 0.85 | 8.42 ± 0.84 | 8.11 ± 0.88 | 8.32 ± 0.87 |
|  | *p*-values | 0.612 | 0.355 | 0.940 | 0.765 | 0.765 |
| Note: *p*-values derived from Wilcoxon Signed Ranks Test. Control to learning night contrasts are of exploratory nature. Note that HRV data for state wake was only available for a limited amount of participants (*n* = 8) and should therefore be interpreted with caution. CPSG = control condition polysomnography; LPSG = learning condition polysomnography. REM = rapid eye movement sleep. RRI = R-R interval; SDNN = standard deviation of R-R intervals; LF = low frequency band (0.04 - 0.15 Hz); HF = high frequency band (0.15 – 0.40 Hz). | | | | | | |

**
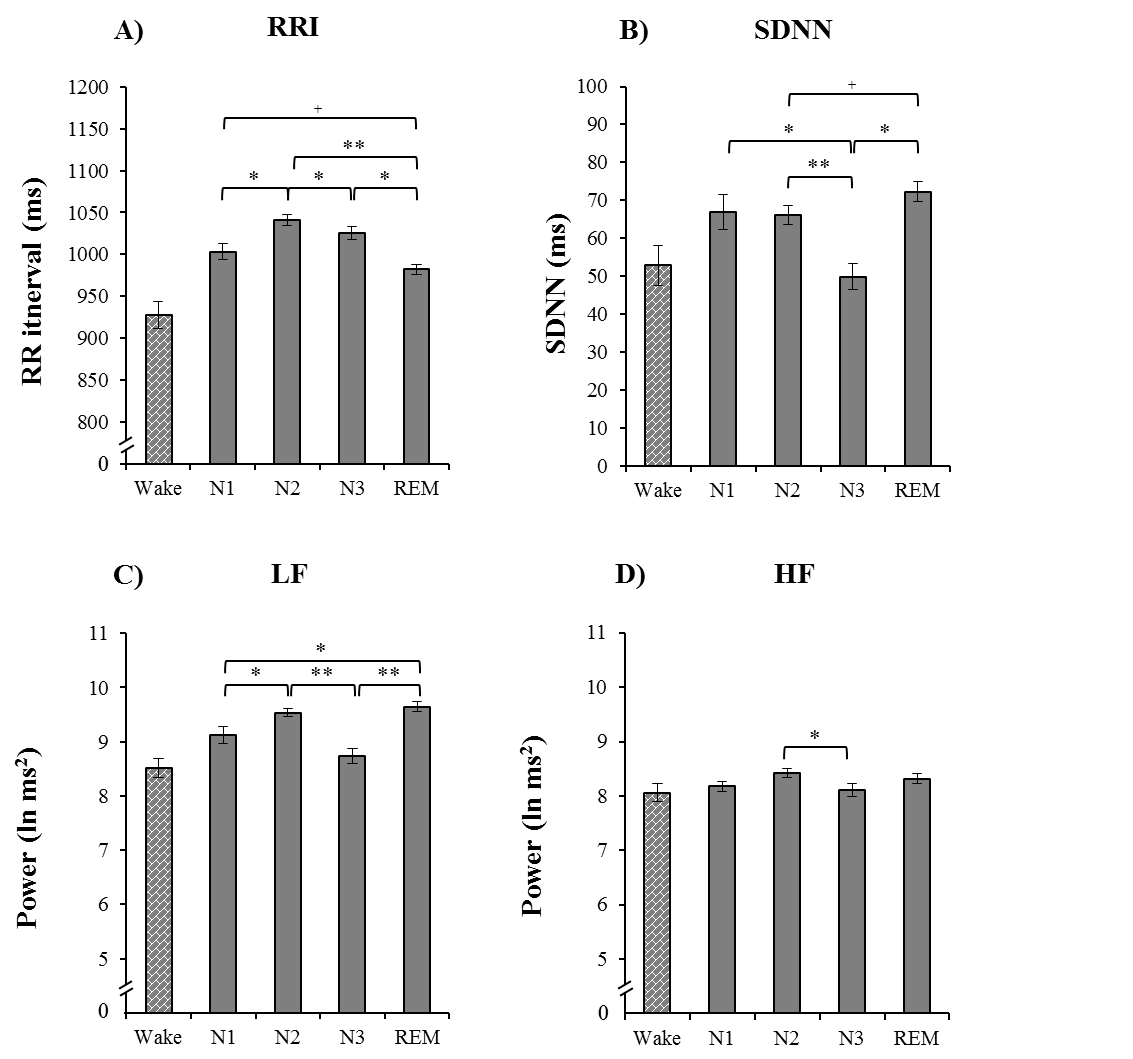
**

**Figure S3. Contrasts between stages for HRV variables during the learning night (LPSG).** Note that error bars have been corrected to illustrate within-subject variability (*M* ± *SE*). Contrasts were conducted for **(A)** R-R interval (RRI), **(B)** standard deviation of R-R intervals (SDNN), and power values in frequency ranges of the low frequency band (LF; 0.04 – 0.15 Hz; **C**) and high frequency band (HF; 0.15–0.4 Hz; **D**). Note that sufficient HRV data for state wake was only available for a limited sample of participants (*n* = 8) and should therefore be interpreted with caution. + *p* <0.10, * *p* <0.05, ** *p* <0.001.

**HRV and overnight performance changes**

Following previous studies (Naji et al., 2019, Whitehurst et al., 2016), associations between OMC and HRV were investigated (Table S3). Overnight memory change for speed was negatively associated with SDNN (*r_s_*[12] = -0.701, *p* = 0.005), LF (*r_s_*[12] = -0.723, *p* = 0.003), and HF (*r_s_*[12] = -0.600, *p* = 0.023) during REM, meaning that higher inter-beat variability and LF power resulted in worsened OMC. LF power during REM was also found to be negatively associated with overnight memory change for the number of errors (*r_s_*[18] = -0.492, *p* = 0.028). Note that no further associations were observed between OMC and HRV measures for NREM sleep stages (all *p* ≥0.052), nor were any associations found between long-term memory changes and HRV measures for any sleep stage (all *p* ≥0.051). Importantly, only the negative association between OMC for speed and LF power during REM remains significant after Bonferroni correction for multiple comparisons (*α* = 0.0042).

| **Table S3**. Spearman correlations between overnight performance changes and HRV during REM and N1 | | | | |
| --- | --- | --- | --- | --- |
|  |  | **Speed**  **OMC** | **Error number**  **OMC** | **Error time (%)**  **OMC** |
| REM | RRI (ms) | 0.059 | -0.078 | 0.208 |
|  | SDNN | -0.701* | -0.370 | -0.118 |
|  | LF (ln ms^2^) | -0.723** | -0.492^+^ | -0.365 |
|  | HF (ln ms^2^) | -0.600^+^ | -0.343 | -0.156 |
| N1 | RRI (ms) | 0.020 | -0.158 | 0.024 |
|  | SDNN | -0.301 | -0.143 | -0.127 |
|  | LF (ln ms^2^) | -0.446 | -0.045 | -0.392 |
|  | HF (ln ms^2^) | -0.486 | -0.441 | -0.427 |
| Note: Overnight memory change (OMC) for speed was found to be negatively correlated with SDNN and LF during REM and N1. OMC = overnight memory change. REM = Rapid Eye Movement sleep. RRI = R-R interval; SDNN = standard deviation of R-R intervals; LF = low frequency band (0.04 - 0.15 Hz); HF = high frequency band (0.15 – 0.40 Hz). Alpha level after Bonferroni correction (*α* = 0.0042). + *p* <0.05, * *p* <0.01, ** *p* <0.0042. | | | | |
